# Measuring influenza laboratory capacity: use of a tool to measure improvements

**DOI:** 10.1186/s12879-017-2521-7

**Published:** 2017-06-15

**Authors:** Pam Kennedy, Tricia Aden, Po Yung Cheng, Ann Moen

**Affiliations:** 1 McKing Consulting Corporation, Atlanta, GA USA; 2 Battelle Memorial Institute, Columbus, OH USA; 3 Centers for Disease Control and Prevention, Atlanta, GA USA; 4 Influenza Division, National Center for Immunization and Respiratory Diseases, U.S. Centers for Disease Control and Prevention, 1600 Clifton Road, Atlanta, GA 30333 USA

**Keywords:** Capacity building, Influenza, Laboratory, Global Health, Surveillance, Assessment

## Abstract

**Background:**

To collect information, identify training needs, and assist with influenza capacity building voluntary laboratory capacity assessments were conducted using a standardized tool in CDC cooperative agreement countries. To understand the usefulness of comparing results from repeat assessments and to determine if targeted training supported improvements, this paper details comparison of assessment results of conducting 17 repeat laboratory assessments between 2009 and 2013.

**Methods:**

Laboratory assessments were conducted by SMEs in 17 laboratories (16 countries). We reviewed the quantitative assessment results of the laboratories that conducted both an initial and follow up assessment between 2009 to 2013 using repeated measures of Anova, (Mixed procedure of SAS (9.3)). Additionally, we compared the overall summary scores and the assessor recommendations from the two assessments.

**Results:**

We were able to document a statistically significant improvement between the first and second assessments both on an aggregate as well as individual indicator score. Within the international capacity tool three of the eight categories recorded statistically significant improvement (equipment, management, and QA/QC), while the other tool categories (molecular, NIC, specimen, safety and virology) showed improvement in scores although not statistically significant.

**Conclusions:**

We found that using a standardized tool and quantitative framework is useful for documenting capacity and performance improvement in identified areas over time. The use of the tool and standard reports with assessor recommendations assisted laboratories with establishing, maintaining, and improving influenza laboratory practices. On-going assessments and the consistent application of the analytic framework over time will continue to aid in building a measurement knowledge base for laboratory capacity.

**Electronic supplementary material:**

The online version of this article (doi:10.1186/s12879-017-2521-7) contains supplementary material, which is available to authorized users.

## Background

The World Health Organization (WHO) Global Influenza Surveillance and Response System (GISRS) has monitored influenza surveillance for over half a century [1]. Since inception of the network in 1952, the WHO has recognized five Collaborating Centres for Reference and Research on Influenza (WHOCC) and more than 140 National Influenza Center (NIC) laboratories [2]. Serving as an important part of the global influenza surveillance and response system, these NICs together with designated national laboratories monitor both current and emerging viruses for the detection of unusual strains and to inform vaccine strain selection [2].

As a precursor to the establishment of the GISRS system, in 1948 WHO members adopted a set of standard regulations to monitor infectious diseases. This initial set of regulations, through subsequent revisions, evolved into the International Health Regulations (2005) (IHR) [3]. The purpose of the IHR (2005) is to prevent, protect, and provide an international public health response to disease [4]. Article 13 (Public Health Response) specifically states requirements for countries to build and maintain capacity to identify and respond to public health risks and emergencies [4]. Laboratory services play a critical role in a country’s ability to respond to public health concerns and are identified in many of the key processes within the IHR (2005) [5]. The Centers for Disease Control and Prevention (CDC) Influenza Division uses bi-lateral cooperative agreements to support capacity development and maintenance of epidemiology and laboratory infrastructure in over 50 countries [6]. More specifically these cooperative agreements provide technical support in laboratory diagnostics, epidemiology, surveillance, preparedness, and communication strategies [7].

To collect information from cooperative agreement countries, voluntary laboratory capacity assessments were conducted using a standardized tool developed by CDC and Association of Public Health Laboratories (APHL) subject matter experts (SMEs). The tool serves as a framework for development of recommendations to guide improvements to laboratory capacity for monitoring seasonal influenza and responding to novel influenza emergencies [8]. The tool’s development process and the addition of a quantitative framework to measure change is previously described [8]. Within the tool, the questions are assigned to eight modules of general categories each representing a different area of the laboratory. These are; general laboratory, specimen handling, virology laboratory, molecular biology, biosafety, quality assurance, equipment and NIC criteria [8].

To understand the usefulness of comparing results from repeat assessments and determine if targeted training supported improvements, this paper details comparison of assessment results for 17 laboratories conducting repeat assessments between 2009 and 2013.

## Methods

Laboratory assessments were conducted by SMEs in 17 laboratories (16 countries). Countries included: Angola, Armenia, Burkina Faso, Democratic Republic of Congo, Fiji, Laos, Mongolia, Nepal, Nicaragua, Nigeria, Paraguay, Rwanda, Sri Lanka, Tanzania, Ukraine and Vietnam where two laboratories were assessed. We reviewed laboratories that conducted an initial and a follow up assessment in the 2009 to 2013 timeframe and documented the time between assessments. Using an Excel spreadsheet format (tool), assessors answered questions and provided comments and observations.

Assessments conducted in the 2009 to 2011 timeframe were documented in an initial version of the tool. In 2011, the tool was improved based on assessor feedback and a quantitative framework was added to determine scores and trends. To eliminate any potential bias, the quantitative framework sheet was hidden from the assessor’s view. Each assessment generated a list of recommendations for the assessed country, designed to assist with overall laboratory techniques, management, and safety improvement.

Four laboratories were assessed twice with the same tool and thirteen of the secondary assessments were conducted with the improved tool that included the quantitative framework. Scoring was retrospectively applied to the original tool version and used for comparison purposes. [8] To verify if the improvement in score was the result of increased capacity and laboratory performance rather than the result of tool changes, tool version was included as one of the factors in the statistical analysis model.

Since a main purpose of this review was to determine if the tool is useful for measuring differences between assessments by area or category, repeated measures of Anova, (MIXED procedure of SAS (9.3)) [9] was used to examine the effect of initial and follow up assessments on the adjusted score (total score divided by the total possible points for each category and each country). This base model included the fixed effect of the interaction between category and assessment. Country was included in the models as a random effect. Model effects were considered statistically significant when a Type I error rate was less than 5%. The corresponding least squares means (lsmeans) and their 95% confidence intervals are presented to facilitate interpretation of results. To test the performance of the ANOVA MIXED procedure with the data that might not have a normal distribution, we did the arcsine square root transformation of the adjusted scores. Then we used these transformed data to run the ANOVA MIXED procedure and compared the results before and after the data transformation. A sensitivity analysis was conducted by excluding four countries that used the same version of the assessment tool for both assessments. The changes of the adjusted scores between first and second assessments for the full 17 laboratories were compared with those for the reduced number of laboratories.

Due to availability of assessors, follow up assessments often necessitated the use of different assessors. Twelve of the 17 secondary assessments were conducted by an assessor different from the initial assessment. To determine if different assessors play a role in affecting the scores, we added a variable “assessor” to the base model to see if this variable was significant or not. To determine effects of time on assessment scores, we first looked at the distribution of the length of time (months) between the two assessments for these 17 laboratories. Because there was a bimodal distribution, we set a dummy variable “length of time” (<27 months or 27+ months) and added it to the base model. We want to see how the length of time plays a role on the outcome. We assume that a longer length of time is needed for observing significant improvement.

Finally, we conducted a comparison of summary scores and assessor recommendations from the two assessments. We added all possible points available during the assessment and actual scores received and divided the score by the total possible points to determine a percentage. This calculation was done both overall and by each category. Assessor recommendations were documented in the final reports. Final reports from each assessment were reviewed to identify recommendations and status updates were obtained during a follow-up assessment to gather information on which of the original recommendations were implemented and which items remained pending. Analysis of the recommendation results is previously discussed [10].

## Results

Comparison of the first and second assessments for 17 laboratories shows statistically significant improvement on the adjusted score when all categories are aggregated for all laboratories (from 0.57 to 0.67, *p* = <.0001) (Table 1). In addition to improvement on an overall aggregate level, three of the eight categories recorded statistically significant improvement (equipment, management, and quality assurance/quality control (QA/QC)). (Table 1) The five remaining categories (molecular, NIC, specimen, safety and virology) showed improvement in scores although not statistically significant. Since the percentage data (adjusted scores) might not have a normal distribution, we did the arcsine square root transformation of the adjusted scores before running the ANOVA MIXED procedure. The results from the transformed data presented the similar pattern in terms of Type-I error (see Additional file 1: Table S1). The sensitivity analysis conducted to compare results of all 17 laboratory assessments to the 4 that were conducted with the same tool showed no effect on scoring when using different versions. In examining the use of different versus same assessors, no statistical significance (*p*-value = 0.6715) was observed nor did the use of different assessors negatively impact the scores. The median time between the initial and subsequent assessment was 2.9 years with a range from 1.2 to 3.7 years. Analysis shows that while there is positive movement for all assessments, there is a statistically significant difference when the time frame between assessments is greater than 27 months (*P*-value <0.0001).

**Table 1:** Statistical Significance between First and Second Assessments on the adjusted scores^a^

Category	First Assessment	95% Confidence Interval		Second Assessment	95% Confidence Interval		*p*-value
		Lower	Upper		Lower	Upper	
Overall Assessment	0.57460	0.49340	0.65570	0.67080	0.58970	0.75720	<.0001
Equipment	0.39220	0.27600	0.50830	0.58780	0.47160	0.70390	0.0003
Management	0.60540	0.48930	0.72160	0.74730	0.62750	0.85980	0.0099
Molecular	0.67910	0.56300	0.79530	0.78070	0.66460	0.80600	0.0564
NIC Criteria	0.50000	0.38380	0.61620	0.53100	0.41490	0.64720	0.5578
QA/QC	0.54800	0.43190	0.66420	0.72610	0.60990	0.84220	0.0010
Safety	0.54590	0.42970	0.66200	0.64780	0.53170	0.76400	0.0556
Specimen	0.77290	0.65670	0.88900	0.79080	0.67470	0.90700	0.7340
Virology	0.55290	0.43680	0.66910	0.55880	0.44270	0.67500	0.9114

^a^Multivariate model with the adjusted score as outcome and the category, the assessment, and its interaction term as independent variables

In comparing the categories and their actual scores over time we found positive gains of 12% overall with positive trends for each category ranging from a gain of 2% to 20% (Table 2). Revisions to the tool between the first and second assessments accounts for the difference in possible points between the two assessments. Table 2 shows aggregate scores for all assessments.

**Table 2:** Comparison of 1st v. 2nd assessments percentage scores

Category	Possible Points	1st Assessment Score	Percentage	Possible Points	2nd Assessment Score	Percentage	Percentage (Gain/Loss)
Overall Total	2076	1183	57%	2323	1607	69%	12%
Equipment	188	74	39%	201	119	59%	20%
Management	228	138	61%	319	241	76%	15%
Molecular	187	127	68%	187	146	78%	10%
NIC Criteria	171	85	50%	184	98	53%	3%
QA/QC	478	262	55%	504	367	73%	18%
Safety	528	288	55%	541	351	65%	10%
Specimen	210	162	77%	288	229	80%	3%
Virology	86	47	55%	99	56	57%	2%

## Discussion

In examining the three categories that showed statistically significant improvement, the equipment category (which focuses on the appropriate maintenance and certification of equipment as well as the overall structure of the laboratory building) showed the most improvement. Laboratories completed recommendations to implement maintenance agreements and annual calibration of equipment as well as implementing regular testing of backup generators to drive the positive improvement. The QA/QC category focuses on processes and procedures designed to minimize error. The management category highlights overall laboratory processes such as inventory control. Those with statistically significant gains appear more procedural in nature. By simply ensuring practices were written into standard operating procedures and made available to staff, laboratories improved both their quality and overall management practices. The five remaining categories: virology laboratory, molecular laboratory, specimen collection, safety and NIC criteria showed improvement, though not statistically significant. These are more dependent on human resources and training. Skills are needed to isolate viruses, perform molecular procedures and specimen collection and these skills take time, human resources and training effort to implement, which may take more time to show significant improvement. The safety category reviews use of personal protective equipment (PPE) and inclusion of safety procedures for laboratory practices. In addition, two categories, molecular biology and specimen collection, were among the highest scoring in the initial assessments and thus statistical improvement is harder to achieve.

To validate that positive improvement was the result of actual improved work processes and procedures and not driven by changes in the tool or with different assessors, we asked assessors to review recommendations from the first assessment and document the status where actual improvements were made. Items such as lack of equipment maintenance agreements, inventory control or ordering procedures, written crisis plans and protocols for testing were examples of recommendations in the initial assessments. During subsequent assessments, these items were addressed or in the process of being completed. In addition, as other examples of progress, laboratories had purchased either equipment maintenance agreements or hired maintenance staff and developed and implemented written protocols for testing and biosafety. A recent article provides detailed analysis of all the recommendations made during the first assessment and the documented status garnered from either a follow up assessment or from laboratory personnel updates [10].

**Fig. 1:**
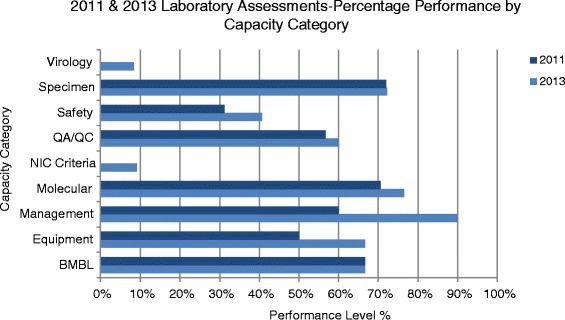
Graphical Representation of Assessment Scores

Recommendations provided necessary information to understand the needs and gaps in each country. In addition, comparing an individual country’s percentages for each category from first to second assessments is useful to identify areas of need. Charts displaying each category’s status for sequential assessments have been incorporated into our standard assessment reports. This provides a quick visual display of gains or losses during subsequent assessments. Lack of a value for a particular year is indicative of a 0% score in category for that year. A representative chart from one country is shown in Fig. 1.

A well-designed tool should identify gaps to assist in developing targeted training programs. By aggregating information from multiple countries within a Region, we were able to identify and assess common regional gaps. The identified gaps assisted with the development of the laboratory training content. For example, data and recommendations from the 2009 to 2011 assessments were aggregated and utilized to develop the curriculum for both laboratory management and biosafety trainings. These trainings included topics such as; “Human Resources Basics, Biosafety for Lab Managers, Quality Assurance and Quality Control, Inventory Management…” The trainings were offered in three different regions around the world in 2011 (South Africa), 2012 (Bangkok), and 2014 (Istanbul). Of the 17 laboratories assessed, 10 (59%) attended one of these laboratory trainings between their first and second assessments. Of the 10 countries attending training 9 out of 10 improved in overall management (quality assurance, inventory management, human resources, standard operating procedures, etc.) scores with 1 country maintaining the same score. The laboratory safety category also had 9 of 10 countries improving with the remaining country slightly decreasing its score. The topics of the training largely match the areas of the tool where subsequent assessments yielded statistically significant improvement overall. While we cannot precisely relate our training to the gains, the documentation of the needs in laboratories followed by our design of training to address these needs is compelling. Trainings to address additional gaps such as PCR and virus isolation are also conducted as needed.

One of the difficulties and a potential limitation in conducting assessments using a common tool with different assessors is the issue of inter rater consistency. Guidance for assessors using the original tool was provided through a webinar that presented the purpose of the tool and assessment and a simple overview with instructions. With the development of the revised tool we sought to address inter rater consistency by creation of a guidance document to minimize any potential issues and support consistency across different time intervals, assessors, and laboratories. The guidance document contains specific instructions and examples to assist with addressing all questions. Assessors were provided training prior to performing assessments with the revised tool including exercises on inter rater consistency. The lack of a significant effect on the use of different assessors versus the same assessor over time suggests the exercises for interrater consistency, guidance documents and training have been effective. Another limitation for this study is the small number of laboratories relative to the number of parameters in the model. The small sample size resulted in the wider confidence intervals in this study.

We were able to document a slight effect of time or length of time between assessments on score variability. Analysis shows that while there is positive movement for all assessments, there is a statistically significant difference when the time frame between assessments is greater than 27 months. This timeframe designation was driven by the available dataset. A limitation identified within this assessment process is that improvement, in large part, is driven by a willingness to improve as well as the infrastructure of the country’s Ministry of Health. To provide time for impact and improvement, the assessments should be conducted far enough apart to allow for implementation of the identified recommendations. As more data are collected, this analysis could be revisited to validate the statistical significance for either a shorter or longer timeframe between assessments. More data points are needed to assess the actual impact of time span on quality improvement. More importantly these data can help us determine optimal timeframes between assessments to maximize use of travel and assessor resources.

While programs strive for continuous improvement, revising our tool to add an analytic framework posed challenges. Performance measurement is a key component for analytic analysis and an applied quantitative framework that facilitates performance measurement should be included as part of the tool development process. By including this component in the development process, current status can be documented and action plans/recommendations for issue resolution can be developed.

## Conclusion

The need to document influenza laboratory performance guided the development of the International Influenza Laboratory Capacity Review Tool. Based on analysis for 17 laboratories, we found that using a standardized tool and quantitative framework is useful for documenting capacity and performance improvement in identified areas over time. Progress was verified by both data and comments recorded by assessors. The format allowed easy visual representation of the status of gains or losses between reviews which is useful for countries and programs as they address needs.

Important to our program, the tool was useful in identifying gaps, and was used to develop targeted laboratory training as well as overall training curriculums. We were able to show statistically significant gains in the areas our curriculum targeted.

There were limitations to the process and the implementation of the tool. The major limitations we identified were the reliance on country availability and engagement in the entire process as well as availability of qualified assessors to conduct reviews. Engagement of the ministries of health in different countries was critical for both the scheduling and for implementation and support for identified improvements. The variability of country support led to variations in overall improvement. Lastly, tool improvement and revision during the process added challenges for comparing results over time. Question consistency, assessment to assessment, is important to ease of data comparison over time.

The use of the tool and standard reports with assessor recommendations assisted laboratories with establishing, maintaining, and improving influenza laboratory practices. On-going assessments and the consistent application of the analytic framework over time will continue to aid in building a measurement knowledge base for laboratory capacity and laboratory-based influenza surveillance; an area that currently has limited available information.

## Additional file

Additional file 1: Table S1. Statistical Significance between First and Second Assessments on the adjusted scores (with the arcsine transformation)*. (DOC 38 kb)

## Abbreviations

WHO: World Health Organization
GISRS: Global Influenza Surveillance and Response System
WHOCC: WHO Collaborating Centers
NIC: National Influenza Center laboratories
IHR: International Health Regulations
CDC: Centers for Disease Control and Prevention
APHL: Association of Public Health Laboratories
SME: Subject matter experts
QA/QC: Quality assurance/quality control
PPE: Personal protective equipment
