# Supplementary material for: Measuring influenza laboratory capacity: use of a tool to measure improvements
Source: BMC Infect Dis. 2017 Jun 15;17:431. doi: 10.1186/s12879-017-2521-7 (PMC5472927; doi:10.1186/s12879-017-2521-7)
Supplement: Additional file 1: Table S1. — Statistical Significance between First and Second Assessments on the adjusted scores (with the arcsine transformation)*. (DOC 38 kb) [file 12879_2017_2521_MOESM1_ESM.doc]

| Supplemental Table 1. Statistical Significance between First and Second Assessments on the adjusted scores (with the arcsine transformation)* | | | | | | | | | |
| --- | --- | --- | --- | --- | --- | --- | --- | --- | --- |
|  |  |  |  |  |  |  |  |  |  |
| Category | First Assessment | 95 % Confidence Interval | |  | Second Assessment | 95 % Confidence Interval | |  | p-value |
|  |  | Lower | Upper |  |  | Lower | Upper |  |  |
| Overall Assessment | 0.8640 | 0.7604 | 0.9675 |  | 0.9817 | 0.8782 | 1.0852 |  | <.0001 |
| Equipment | 0.6701 | 0.5174 | 0.8228 |  | 0.8910 | 0.7382 | 1.0437 |  | 0.0017 |
| Management | 0.8838 | 0.7311 | 1.0365 |  | 1.0569 | 0.9042 | 1.2096 |  | 0.0131 |
| Molecular | 0.9929 | 0.8402 | 1.1456 |  | 1.1207 | 0.9680 | 1.2735 |  | 0.0654 |
| NIC Criteria | 0.7584 | 0.6057 | 0.9111 |  | 0.8211 | 0.6684 | 0.9738 |  | 0.3636 |
| QA/QC | 0.8357 | 0.6829 | 0.9884 |  | 1.0377 | 0.8850 | 1.1905 |  | 0.0039 |
| Safety | 0.8382 | 0.6855 | 0.9909 |  | 0.9437 | 0.7910 | 1.0964 |  | 0.1277 |
| Specimen | 1.1047 | 0.9520 | 1.2574 |  | 1.1194 | 0.9667 | 1.2721 |  | 0.8311 |
| Virology | 0.8279 | 0.6752 | 0.9806 |  | 0.8631 | 0.7103 | 1.0158 |  | 0.6103 |
| *Multivariate model with the adjusted score (with the arcsine transformation) as outcome and the category, the assessment, and its interaction | | | | | | | | | |
|  | | | | | | | | |  |
